# Who should receive treatment? An empirical enquiry into the relationship between societal views and preferences concerning healthcare priority setting

**DOI:** 10.1371/journal.pone.0198761

**Published:** 2018-06-27

**Authors:** Vivian Reckers-Droog, Job van Exel, Werner Brouwer

**Affiliations:** 1 Erasmus University Rotterdam, Erasmus School of Health Policy & Management, Rotterdam, the Netherlands; 2 Erasmus University Rotterdam, Erasmus School of Economics, Rotterdam, the Netherlands; Universidad del Desarrollo, CHILE

## Abstract

**Introduction:**

Policy makers increasingly need to prioritise between competing health technologies or patient populations. When aiming to align allocation decisions with societal preferences, knowledge and operationalisation of such preferences is indispensable. This study examines the distribution of three views on healthcare priority setting in the Netherlands, labelled “Equal right to healthcare”, “Limits to healthcare”, and “Effective and efficient healthcare”, and their relationship with preferences in willingness to trade-off (WTT) exercises.

**Methods:**

A survey including four reimbursement scenarios was conducted in a representative sample of the adult population in the Netherlands (n = 261). Respondents were matched to one of the three views based on their agreement with 14 statements on principles for resource allocation. We tested for WTT differences between respondents with different views and applied logit regression models for examining the relationship between preferences and background characteristics, including views.

**Results:**

Nearly 65% of respondents held the view “Equal right to healthcare”, followed by “Limits to healthcare” (22.5%), and “Effective and efficient healthcare” (7.1%). Most respondents (75.9%) expressed WTT in at least one scenario and preferred gains in quality of life over life expectancy, maximising gains over limiting inequality, treating children over elderly, and those with adversity over those with an unhealthy lifestyle. Various background characteristics, including the views, were associated with respondents’ preferences.

**Conclusions:**

Most respondents held an egalitarian view on priority setting, yet the majority was willing to prioritise regardless of their view. Societal views and preferences concerning healthcare priority setting are related. However, respondents’ views influence preferences differently in different reimbursement scenarios. As societal views and preferences are heterogeneous and may conflict, aligning allocation decisions with societal preferences remains challenging and any decision may be expected to receive opposition from some group in society.

## Introduction

Healthcare resources are scarce and policy makers in publically funded healthcare systems are increasingly confronted with the need to prioritise between competing health technologies or patient populations for reimbursement [1, 2]. An important objective of a healthcare system is to generate as much health as possible, given the budget constraint [3]. To achieve this objective, economic evaluations of (new) health technologies are applied to guide policy makers in making decisions concerning the allocation of healthcare resources [4, 5]. In health-economic evaluations, the value of a health technology is commonly expressed in terms of quality-adjusted life-years (QALYs) and evaluated against some monetary threshold value per QALY gained [1, 6].

Regardless of whether the decision rule for economic evaluations implies the maximisation of health under a fixed budget or the maximisation of welfare for society, traditionally, health technologies with lower incremental costs per QALY ratios (ICERs) than some relevant threshold are eligible for funding [3, 4, 7]. Often, both the weight attached to QALY gains and the applied thresholds are constant in such evaluations. This assumes that “a QALY is a QALY is a QALY”, regardless of beneficiary characteristics and the context in which QALYs are gained [8]. However, the practice of valuing all QALY gains equally, and hence regardless of these aspects, has become a matter of debate as evidence is accumulating that this may insufficiently reflect societal preferences [1, 4, 6, 9–12]. Indeed, the public also considers an equitable or fair allocation of health and healthcare important in the allocation of healthcare resources [4, 10, 13, 14] and societal preferences concerning healthcare priority setting are related to the (1) characteristics of healthcare beneficiaries, e.g. a patient’s age, potential to benefit from treatment, remaining life-years, social role, and lifestyle, (2) characteristics of the disease, e.g. the rarity of a disease and the burden of illness associated with a disease prior to treatment, and (3) characteristics of interventions, e.g. the size, type, duration, and costs of health gains [1, 4, 10, 15–18]. Although health economists tend to agree that such preferences should play a role in decisions concerning resource allocation in healthcare [4], they are generally not included in health-economic evaluations (even though notable exceptions like in the Netherlands exist [19]). The discrepancy between prioritisation based on health-economic evaluations and societal preferences for distributing health and healthcare is considered one of the reasons for the modest impact of health-economic evaluations on the outcome of allocation decisions [20–22]. To bridge this gap, knowledge and operationalisation of an equity-dependent decision rule appears to be indispensable.

Empirical evidence suggests that, although some members of the public appear unwilling to prioritise in healthcare, the majority accepts priority setting as being necessary [4, 18, 23]. However, little is known about the criteria that should be used according to the public and about the weight these should receive in allocation decisions [17, 18, 24–27]. Commonly, studies examine societal preferences for priority setting on an aggregate or mean level [28]. Less common are studies that examine the heterogeneity of societal preferences or the relationship between underlying rationales and preferences [17, 18, 25–27]. In a previous study, Wouters et al. [17] used Q methodology to identify three societal viewpoints regarding healthcare priority setting among members of the public in the Netherlands: “Equal right to healthcare”, “Limits to healthcare”, and “Effective and efficient healthcare”. These views are described in Box 1. In the current study, we examine the distribution of the three views in the general adult population and the relationship between these views and preferences concerning healthcare priority setting in four willingness to trade-off (WTT) exercises to inform priority-setting decisions in healthcare.

**Box 1. Societal viewpoints on healthcare priority setting in the Netherlands.**

The view “Equal right to healthcare” comprises an egalitarian view on health and healthcare. People with this view consider access to healthcare a basic human right. Everyone is equal, hence has an equal right to healthcare. According to people with this view, prioritisation should solely be based on the need for care and prioritisation based on patient, disease, and intervention characteristics, such as the effect of treatment, is opposed. What is considered to be “the right care” is a matter of personal concern for patients and, according to people with this view, patients should be supported in their treatment choices regardless of the costs.

The view “Limits to healthcare” comprises a view with a strong concern for providing “the right care” for patients. People with this view consider health-related quality of life to be an important outcome of treatment. According to people with this view, providing the right care may imply refraining from (life prolonging) treatment. People with this view do not consider cost-effectiveness to be an important criterion for priority setting, although they do consider it important to make good use of money. Hence, providing treatments that generate minimal benefits should be avoided. Priority setting based on patient characteristics is rejected, with an exception made for lifestyle. According to people with this view, patients who are culpable of their own disease should receive lower priority and prevention should receive higher priority in allocation decisions.

The view “Effective and efficient healthcare” comprises a utilitarian view on health and healthcare. People with this view consider it important to generate as much health for society as possible given the budget constraint, and consider a patient’s capacity to benefit from treatment important when setting priorities. Although people with this view focus on the cost-effectiveness of treatments, they do believe it is not possible to “put a [fixed] price on life”. The value of health benefits depends on circumstances and patient characteristics, such as age and culpability, and hence these should be taken into account in priority setting.

Note: A detailed description and a discussion of the three viewpoints can be found in Wouters et al. (2015).

## Methods

### Sample and data collection

A professional internet survey company in the Netherlands distributed the questionnaire in October and November 2015, to a random sample that was stratified in terms of age, gender, and education level in order for it to be representative of the general adult population in the Netherlands regarding those characteristics. According to the Medical Research Involving Human Subjects Act, no ethical approval was required for this study. Prior to participating in the study, respondents were informed about the objectives of the study and how anonymity of respondents was guaranteed. They were informed that participation in the study was voluntary and could be stopped at any time, in which case the data they had provided would be discarded. Respondents could only enter the study after giving written consent for the use of their data for the purpose of the study.

Before answering questions about distributive preferences, respondents were explained that healthcare resources are scarce and that health policy makers inevitably have to make difficult choices between competing health technologies or patient populations for reimbursement. It was explained that the consequence of reimbursing one (type of) technology for one patient group implied not being able to reimburse another. Subsequently, respondents were asked to advise health policy makers on what would be the optimal allocation of available healthcare resources in four reimbursement scenarios.

In the next subsection, the questionnaire, including the statements and reimbursement scenarios, that was used for matchings respondents to a view and for eliciting their preferences is described. Subsequently, the reimbursement scenarios, scenario characteristics, and accompanying WTT exercises are discussed in more detail. In the final subsection of the Methods section, the analyses and hypotheses are described that were used for examining the distribution of the views and the relationship between respondents’ views and preferences concerning healthcare priority setting.

### Questionnaire

The questionnaire consisted of three parts. In part one, respondents were asked about demographic and background characteristics. In part two, respondents were asked to express their level of agreement on a seven-point Likert scale (ranging from completely disagree to completely agree) with 14 statements on principles for resource allocation that were extracted from Wouters et al. [17] and presented to respondents in random order. Table 1 presents these statements including respondents’ mean (SD) level of agreement with each of the statements. To match respondents to one of the three views on healthcare priority setting, four statements were selected for each of the views and two additional statements were selected to untie, in case a respondent scored similarly on more than one view. The statements were selected based on the criteria that a statement should be characterising and distinguishing for one of the three views, which means that the statement should have a high factor score in that view and/or that this score should be statistically significantly different from factor scores of the other two views [17, 29, 30]. The assumption underlying the matching of respondents to one of the views was that respondents who expressed a relatively high level of agreement with statements that are characteristic and/or distinguishing for a specific view have a view that is similar to that view.

**Table 1:** Overview of statements used for matching respondents to one of three societal views on healthcare priority setting (weighted data, n = 261).

				Factor score^a^			
View	#	Statement		F1	F2	F3	Mean (SD)^b^
Equal right to healthcare	1	If it is possible to save a life, every effort should be made to do so		+3*	0	-2	5.21 (1.57)
	2	If there is a way of helping patients, it is morally wrong to deny them this treatment		+3*	+1	+1	5.38 (1.42)
	3	It’s important to respect the wishes of patients who feel they should take every opportunity to extend their life		+1*	-3	-1	4.90 (1.35)
	4	Patient characteristics other than their health should play no role in prioritising care		+3*	0	-1	5.18 (1.46)
Limits to healthcare	5	At the end of life it is more important to provide a death with dignity than treatments that will only extend life for a short period of time		+2	+4*	+2	5.17 (1.50)
	6	People should accept that if it’s your time to die, it’s your time to die		0	+3*	0	4.48 (1.64)
	7	People who are in some way responsible for their own illness should receive lower priority than people who are ill through no fault of their own		-2*	+2*	0*	3.49 (1.70)
	8	There is no sense in saving lives if the quality of those lives will be really bad		0	+4*	-2	4.24 (1.63)
Effective and efficient healthcare	9	Children’s health should be given priority over adult’s health		-1*	-3*	+4*	3.78 (1.58)
	10	Priority should be given to patients who benefit most from treatment		-1*	+1*	+4*	4.07 (1.58)
	11	Priority should be given to those treatments that generate the most health		+1	+1	+3*	4.35 (1.57)
	12	Treatments that are very costly in relation to their health benefits should be withheld		-2*	0*	+1*	3.03 (1.56)
Additional statements	13	Treating people at the end of life is important, even if it is not going to result in big health gains		+1*	-1	-3	4.85 (1.50)
	14	Treating terminally ill patients as more ‘worthy’ of receiving care undervalues the health of other patients		0*	-1*	+1*	3.33 (1.67)

^a^ Factor scores and p-values are extracted from Wouters et al. (2015); factor scores range from -4 (disagree most) to +4 (agree most)

* p-value < 0.01; F1 relates to the view “Equal right to healthcare”, F2 to the view “Limits to healthcare”, and F3 to the view “Effective and efficient healthcare”.

^b^ Respondents’ mean (SD) level of agreement with the statements, expressed on a seven-point Likert scale ranging from 1 (completely disagree) to 7 (completely agree).

In part three of the questionnaire, respondents were presented four reimbursement scenarios. The scenarios were based on the study by Wouters et al. [17] and designed in such a way that differences in preferences between respondents with different views could manifest themselves. Each of the scenarios included two options, labelled “A” and “B”, that differentiated two competing treatments, based on the type of health gain, or patient groups, based on patients’ potential to benefit from treatment, age, or lifestyle. Respondents were asked to advise health policy makers on reimbursement, by first choosing between the two treatments or patient groups and subsequently, depending on the scenario, indicating the relative size of the health gain or patient group that would make them indifferent between the two options. Respondents were allowed to opt out in case they had no preference for one of the options. When a respondent chose to opt out, they were asked to explain their choice by checking one of two provided answer options that indicated equality between the treatments or patient groups, e.g. “both treatments are equally effective” or “both treatments are equally ineffective”, or by completing an open text field. As an example, scenario one is included in the supporting information.

### Reimbursement scenarios

The reimbursement scenarios were similarly structured but differed in terms of treatment and patient characteristics. In scenario 1, respondents were asked to choose between two treatments based on their preference for health gains in terms of 3 points in health-related quality of life (QOL) or 3 months in life expectancy (LE), while both patient groups currently had a remaining LE of 3 months with a QOL of 3 points. The QOL scale ranged from 0 to 10, with ‘0’ representing the worst imaginable health state and ‘10’ representing the best imaginable health state. When a respondent preferred treatment A with a gain in QOL, they were asked at which point they would be indifferent between a gain in QOL between 0 and 3 points and a gain of 3 months in LE for treatment B (and vice versa if respondents preferred the LE gain). In scenario 2, respondents were asked to choose between two patient groups based on patients’ potential to benefit from treatment. Respondents stated their preference for maximising health gains or limiting health inequality between the patient groups, by choosing between a 3 point gain in QOL in patient group A or a 1 point gain in QOL in patient group B, while both groups currently had a QOL of 5 (on a scale from 0 to 10). When a respondent preferred the health maximising option, they were asked at which point they would be indifferent between a gain in QOL between 0 and 3 points and a gain of 1 point in QOL for the other patient group (or how large the difference should be, up to 5 points, to switch to patient group A, if they had a preference for patient group B). In scenario 3, respondents were asked to prioritise a 12 month increase in LE by choosing between two patient groups based on their preference for treating children (<18 years) or elderly (>70 years). When a respondent chose to treat the group of children, they were asked at how many months between 0 and 12 months gain in LE they would be indifferent between treatment of the two age groups (and vice versa if respondents preferred to treat the group of elderly). In scenario 4, respondents were asked to choose between two patient groups based on their preference for reducing the risk from 1:1,000 to 1:10,000 of a life-threatening illness for those with an unhealthy lifestyle or those with running the same risk due to adversity (explained to respondents as a reduction from 10 to 1 patients in a population of 10,000). When respondents preferred the patients running the risk due to adversity, they were asked to indicate how many patients between 1 and 10 would make them indifferent between the two groups (and vice versa). The scenarios stated there were no other differences between the treatments or patient groups than the ones described.

In all but scenario 2, the post-treatment health status was equal for patient groups in both options. However, in scenario 1, patients’ post-treatment health status was not measured on a single scale, as in the other scenarios, but on a combined QOL and LE scale. In this scenario, the post-treatment health status was 18 for both patient groups. This was calculated in option A by multiplying 3 months LE by 6 points QOL and in option B by multiplying 6 months LE by 3 points QOL. Although in scenario 1 respondents’ preferences were elicited on a combined QOL and LE scale, respondents who expressed a preference for a gain in QOL indicated their point of indifference on a QOL scale, while those who expressed a preference for a gain in LE indicated their point of indifference on a LE scale. Scenarios 2, 3, and 4 elicited respondents’ point of indifference between the options on a single scale, either in terms of QOL, LE, or in number of patients.

### Statistical analyses and hypotheses

To improve our sample’s representativeness of the general adult population in the Netherlands, we weighted the data by applying a combined weighting factor for age, gender, and education level. For the analyses, respondents were divided into a “traders” and a “non-traders” subsample. Respondents who expressed WTT in at least one of the four scenarios were classified as “trader” and those who did not express WTT were classified as “non-trader”. Demographic and background characteristics of the sample and the two subsamples were calculated in percentages of total and mean (SD). To match respondents to one of the views identified by Wouters et al. [17], we applied the following procedure. First, respondents’ levels of agreement with the four statements were summed for each of the views and, for ease of interpretation, rescaled to a 0–10 scale. Next, respondents were matched to the view with the highest sum score on the condition that this score was above 5.0, hence indicated agreement. When two or three views received an equal highest sum score, the levels of agreement with statements 13 and 14 (see Table 1) were used to untie the scores and, if possible, used to match respondents to one of the views. Differences in characteristics between respondents who could and could not be matched, between traders and non-traders, and between respondents with different views were examined using independent t-, analysis of variance (ANOVA), and Fisher’s exact tests. A Bonferroni correction was applied to adjust for the increased risk of a Type 1 error, caused by multiple comparisons.

In each scenario, the WTT of respondents between treatments or patient groups was examined by calculating the percentage of traders and non-traders, and the median (interquartile) range of indifference points of traders with a preference for option A or B. Differences in WTT and in median indifference points of traders with different views were examined using Fisher’s exact and Kruskal-Wallis tests (Bonferroni corrected). Reasons of non-traders for opting out were explored qualitatively. To relate respondents’ preferences to background characteristics, including view, logit regression models were applied. First, an overall model was composed for the four scenarios. This model included the variables age, age squared (to account for non-linearity), gender, education level, having children, daily smoking, and view. Having children and daily smoking were included as these variables were expected to be associated with the outcomes of interest in scenario 3 and 4. Subsequently, we applied likelihood ratio tests (LRT) to examine if this overall model could be improved for specific scenarios by including additional variables that might also be associated with the outcomes of interest, such as excessive alcohol use and being religious. We used generalised variation inflation factors (VIFs) to examine if the coefficient estimates’ standard errors were inflated by multicollinearity.

Based on the description of the views in Wouters et al. [17], three hypotheses were formulated for the relationship between respondent’s views and preferences concerning healthcare priority setting:

Hypothesis 1: Respondents with the view “Equal right to healthcare” have a lower WTT in all scenarios than respondents with the views “Limits to healthcare” and “Effective and efficient healthcare”.
Hypothesis 2: The view “Limits to healthcare” is positively associated with respondents’ WTT in all scenarios. In addition, respondents with this view express a preference for health gains in terms of QOL in scenario 1, for health maximisation in scenario 2, and for treating those with adversity in scenario 4.
Hypothesis 3: The view “Effective and efficient healthcare” is positively associated with respondents’ WTT in all scenarios. In addition, respondents with this view express a preference for health maximisation in scenario 2, for treating children in scenario 3, and for treating those with adversity in scenario 4.

The analyses were conducted using IBM SPSS Statistics 23.0 (SPSS, Inc, Chicago, Ill., USA) and Rstudio 0.99.903 (Rstudio, Inc., Boston, MA, USA).

## Results

The data was weighted by applying a combined weighting factor for age, gender, and education level with a mean (SD) of 1.00 (0.47). Table 2 presents the descriptive statistics and the distribution of the views in the weighted sample (n = 261), and in the traders and non-traders subsamples. The majority of respondents (n = 198; 75.9%) expressed WTT in at least one scenario. Of the respondents, 90.2% (n = 235) could be matched to one of the views based on their level of agreement with the 12 statements and 3.9% (n = 10) could be matched based on their level of agreement with the two additional statements. A t-test revealed that respondents who could not be matched to one of the views were relatively younger than those who could be matched. Mean (SD) age of respondents who could not be matched was 32.3 (13.8) years and of those could be matched 47.2 (14.7) years. This difference was significant at the 0.01 level (two-tailed, Bonferroni corrected, α/12). In addition, a Fisher’s exact test revealed that the difference in nationality between respondents who could and could not be matched was also significant at the 0.01 level (two-tailed, Bonferroni corrected, α/12). Respondents with a Dutch nationality could more frequently be matched to a view than respondents with a different nationality.

**Table 2:** Sample characteristics (weighted data, n = 261)^a^.

	Total (n = 261)		Traders (n = 198)		Non-traders (n = 63)		p-value
	%	Mean (SD)	%	Mean (SD)	%	Mean (SD)	
Age (Years)		46.3 (15.1)		47.2 (15.1)		43.5 (14.9)	0.087
Gender (Female)	49.4		52.7		44		0.312
Nationality (Dutch)	88.9		88.4		90.5		0.008
Education level^b^							0.003*
Low	23.9		19		39.4		
Middle	50.7		52.6		44.7		
High	25.4		28.5		15.9		
Living situation							0.023
Single	27		24.6		34.6		
Married/living together	63		67.3		49.216.2		
With parents/family or commune/dormitory	9.5		7.3				
Children (Yes)	60.1		64.3		46.5		0.012
Lifestyle							
Smoking (Daily)	17.1		12		32.9		0.001*
Alcohol usage (Excessive)^c^	20.5		21.3		17.9		0.717
Chronic condition (Yes)							
Physical	31.7		31.4		32.8		0.551
Mental	4.6		5		3.4		
Physical and mental	2.7		3.5		0		
Religious (Yes)^d^	26.8		27.2		25.4		0.871
View on healthcare priority setting							0.000***
Equal right to healthcare	64.5		60.1		78.3		
Limits to healthcare	22.5		28.8		2.9		
Effective and efficient healthcare	7.1		7.6		5.4		
Not matched	5.9		3.5		13.4		
Health status (VAS 0–10)		6.8 (1.5)		6.8 (1.6)		7.0 (1.4)	0.337
Happiness (VAS 0–10)		7.2 (1.6)		7.2 (1.6)		7.2 (1.8)	0.905

VAS, Visual Analogue Scale.

^a^ In this table, respondents who expressed willingness to trade-off (WTT) in at least one reimbursement scenario are classified as “traders”, respondents who expressed no WTT in all four reimbursement scenarios are classified as “non-traders”.

^b^ Low = lower vocational and primary school, Middle = middle vocational and secondary school, High = higher vocational and academic education.

^c^ Applied standard for excessive alcohol use for female respondents: consumption of ≥7 alcohol units per week or of ≥4 alcohol units on one day, for male respondents: consumption of ≥14 alcohol units per week or ≥ 6 alcohol units on one day.

^d^ Operationalised by the question “Do you consider yourself to be part of a religious community (yes/no)?”.

* p-value < 0.05

*** p-value < 0.001 (two-tailed, Bonferroni corrected, α/13).

The majority of respondents was matched to the view “Equal right to healthcare” (64.5%), followed by “Limits to healthcare” (22.5%), and “Effective and efficient healthcare” (7.1%). A minority of respondents (5.9%) could not be matched. A similar distribution of views was observed among traders (60.1%, 28.8%, 7.6, and 3.5% respectively). However, among non-traders the view ‘Equal right to healthcare” was considerably more prevalent (78.3%), while the views “Limits to healthcare” and “Effective and efficient healthcare” were less prevalent (2.9% and 5.4%, respectively), and relatively more non-traders could not be matched (13.4%). A Fisher’s exact test (two-tailed, Bonferroni corrected, α/13) revealed that the difference in views between traders and non-traders was significant at the 0.001 level. In addition, a Fisher’s exact test (two-tailed, Bonferroni corrected, α/13) revealed that traders were more frequently highly educated and less frequently smoked daily (p-value<0.05) than non-traders. No differences were revealed between traders and non-traders concerning other characteristics. Between respondents with different views, a Fisher’s exact test (two-tailed, Bonferroni corrected, α/12) revealed a significant difference at the 0.05 level for education level (not in table). Respondents with the view “Equal right to healthcare” were more frequently lower educated than respondents with the views “Limits to healthcare” and “Effective and efficient healthcare”.

Table 3 presents the scenario specifications, the proportion of respondents who were willing to trade-off, respondents’ preferences for option A or B, and their median (IQR) indifference points for each of the scenarios. Although the distribution of indifference points is different between traders with a preference for option A or B, an overlap of IQR can be seen in scenario’s 3 and 4. Note that for scenario 1 the IQR for option A and B are on a different scale.

**Table 3:** Respondents’ willingness to trade-off (WTT) in n and % of total, scenario (S) and option (A and B) specifications, and traders’ preferences for option A and B in % of total and median (IQR) indifference point (weighted data, n = 261).

S	WTT n (%)	Option	Specification	Pre-treatment health status			Treatment benefit			Post-treatment health status^a^		Preference (%)	Median (IQR) indifference point
				QOL^b^	LE^c^	Risk	QOL^b^	LE^c^	Risk	QOL^b^	LE^c^		
1	132 (50.8)	A	QOL	3	3	NS	3	NS	NS	6	3	80.3	1.5 (1.0–2.0)
		B	LE	3	3	NS	NS	3	NS	3	6	19.7	2.0 (1.0–2.5)
2	109 (42.0)	A	Maximize health	5	NS	NS	3	NS	NS	8	NS	91.7	2.0 (1.5–2.0)
		B	Limit inequality	5	NS	NS	1	NS	NS	6	NS	8.3	4.0 (3.9–4.5)
3	123 (47.1)	A	Children	NS	NS	NS	NS	12	NS	NS	12	94.3	6.0 (4.0–10.0)
		B	Elderly	NS	NS	NS	NS	12	NS	NS	12	5.7	9.3 (6.0–10.0)
4	117 (44.8)	A	Lifestyle-related risk	NS	NS	1:1,000	NS	NS	1:10,000	NA	NA	10.3	7.0 (5.5–7.6)
		B	Adversity	NS	NS	1:1,000	NS	NS	1:10,000	NA	NA	89.7	5.0 (4.0–8.0)

IQR = interquartile range; NA = Not Applicable; NS = Not Stated, i.e. equal for both options.

^a^ Post-treatment health status is calculated by aggregating patients’ pre-treatment health status and the treatment benefit.

^b^ Health-related quality of life (QOL) is noted in points on a 0–10 scale.

^c^ Life expectancy (LE) is noted in months.

The percentage of respondents who were willing to trade-off ranged between 42.0% and 50.8% in the four scenarios. The highest WTT percentage was expressed in scenario 1, where respondents were asked to prioritise between health gains in terms of QOL or LE. The lowest WTT percentage was expressed in scenario 2, where respondents were asked to prioritise between maximising health gains and limiting health inequality between patient groups. Of the traders, a large majority expressed a preference for health gains in terms of QOL (80.3%) over gains in LE (19.7%), for maximising health gains (91.7%) over limiting health inequality (8.3%), for treating children (94.3%) over treating elderly (5.7%), and reducing risk for those with adversity (89.7%) over those with an unhealthy lifestyle (10.3%). In each of the scenarios, 81.0% to 84.0% of non-traders consisted of respondents with the view “Equal right to healthcare”, who opted out more frequently than respondents with the views “Limits to healthcare” and “Effective and efficient healthcare”. Table 4 presents the differences in WTT between respondents with different views in each of the scenario. Fisher’s exact tests revealed that these differences were significant at the 0.001 level in scenario 1, 3, and 4, and at the 0.01 level in scenario 2 (two-tailed, Bonferroni corrected, α/4). The difference in the median indifference points of respondents with different views was not significant.

**Table 4:** Frequencies of willingness to trade-off (WTT) of respondents with different views on healthcare priority setting in the four reimbursement scenarios (weighted data, n = 246)^a^.

View	WTT in scenario^b^				No WTT in any scenario
	1	2	3	4	
Equal right to healthcare	74	59	64	58	49
Limits to healthcare	45	36	42	43	2
Effective and efficient healthcare	10	11	11	13	3
n	129	106	117	114	62

^a^ Respondents who could not be matched to one of the views (n = 15) are excluded from this analysis.

^b^ In scenario 1, respondents expressed WTT by choosing for a gain in quality of life or in life expectancy or expressed no WTT by opting out. In scenario 2, respondents expressed WTT by choosing for health maximization or limiting health inequality or expressed no WTT by opting out. In scenario 3, respondents expressed WTT by choosing for treating children or elderly or expressed no WTT by opting out. In scenario 4, respondents expressed WTT by choosing for treating those with an unhealthy lifestyle or adversity or expressed no WTT by opting out. Respondents who opted out in all four scenarios are included in the table under “no WTT in any scenario”. The presented differences in WTT frequencies are significant at the 0.001 level in scenario 1, 3, and 4, and at the 0.01 level in scenario 2 (two-tailed, Bonferroni corrected, α/4).

Between 79.8% and 92.9% of non-traders explained their preference for opting out by checking one of the provided answer options, the remainder by completing the open text field. In scenario 1, 20.2% (n = 26) of the non-traders completed the open text field of which 69.2% stated that the choice between options A and B was not theirs but only for patients themselves to make. For example, because “having a preference for quality of life or life expectancy is a personal matter”. Other explanations for opting out included “both options are very much alike” or “both patient groups will die regardless of treatment”. In scenario 2, 10.9% (n = 16) completed the open text field. Explanations for opting out included “I do not see a difference between the two options”, “I would treat whoever came first”, “the value of a person’s life cannot solely be determined based on the physical condition of that person”, and “quality of life is an abstract concept and it provides too little information to form an informed opinion”. In scenario 3, 7.1% (n = 10) completed the open text field. Explanations for opting out included “although I have a preference for treating children, the age of patients should not matter”, “my preference in this matter depends entirely on the burden of illness of the patients”, and “quality of life matters more than life expectancy”. In scenario 4, 8.7% (n = 12) completed the open text field and stated, for example, “it is nearly impossible to determine whether a person is culpable of their own disease”, “two-third of all cancer cases are caused by having bad luck”, “having an unhealthy lifestyle may be involuntary”, and “having an unhealthy lifestyle is often due to adversity”. The explanations for opting out did not seem to differ between respondents with different views on healthcare priority setting.

Tables 5 and 6 present the results of the logit regression models examining the relationship between background characteristics and the WTT, and the most preferred option of respondents in each of the scenarios, with the baseline set to a preference for opting out.

**Table 5:** Impact of characteristics on the willingness to trade-off (WTT) yes/no of respondents in four reimbursement scenarios (logit regression model, weighted data, n = 246)^a^.

	Scenario 1^b^		Scenario 2^c^		Scenario 3^d^		Scenario 4^e^	
	B (SE)	OR (95% CI)	B (SE)	OR (95% CI)	B (SE)	OR (95% CI)	B (SE)	OR (95% CI)
Age	0.050 (0.061)	1.051 (0.934,1.187)	-0.078 (0.059)	0.925 (0.823,1.039)	-0.168** (0.063)	0.845 (0.745,0.955)	-0.131* (0.062)	0.877 (0.776,0.988)
Age squared	-0.000 (0.001)	1.000 (0.998,1.001)	0.001 (0.001)	1.001 (0.999,1.002)	0.002* (0.001)	1.002 (1.000,1.003)	0.001· (0.001)	1.001 (1.000,1.003)
Female	0.042 (0.284)	1.042 (0.598,1.823)	0.197 (0.286)	1.218 (0.695,2.142)	0.439 (0.298)	1.551 (0.867,2.799)	-0.038 (0.297)	0.962 (0.537,1.723)
Education level (low = reference) Middle	0.560 (0.355)	1.750 (0.879,3.555)	0.267 (0.370)	1.306 (0.635,2.732)	0.442 (0.384)	1.556 (0.738,3.351)	0.413 (0.384)	1.511 (0.717,3.245)
High	1.122** (0.435)	3.072 (1.323,7.325)	0.315 (0.434)	1.371 (0.586,3.232)	1.053* (0.457)	2.865 (1.184,7.155)	0.368 (0.452)	1.445 (0.596,3.531)
Children (no = reference)	0.015 (0.350)	1.015 (0.511,2.022)	-0.199 (0.353)	0.820 (0.406,1.632)	-1.345*** (0.397)	0.261 (0.116,0.555)	-0.377 (0.370)	0.686 (0.328,1.406)
Smoking (not daily = reference)	-0.586 (0.381)	0.557 (0.258,1.158)	-1.164** (0.444)	0.312 (0.122,0.713)	-1.216** (0.453)	0.297 (0.115,0.691)	-1.520** (0.471)	0.219 (0.080,0.522)
View (Equal right to healthcare = reference) Limits to healthcare	1.196** (0.376)	3.306 (1.614,7.102)	1.205*** (0.356)	3.336 (1.677,6.793)	1.641*** (0.398)	5.161 (2.421,11.606)	1.766*** (0.386)	5.850 (2.807,12.843)
Effective and efficient healthcare	0.337 (0.546)	1.401 (0.475,4.158)	0.959· (0.546)	2.608 (0.903,7.901)	0.931 (0.578)	2.536 (0.821,8.150)	1.476* (0.587)	4.375 (1.448,15.043)
Constant	-2.353 (1.622)	0.095 (0.004,2.179)	1.447 (1.595)	4.252 (0.188,100.414)	4.652** (1.717)	104.790 (3.820,3294.049)	2.830· (1.657)	16.950 (0.679,460.767)
AIC	338.81		338.43		311.72		324.33	
R^2^ (Mc Fadden)	0.172		0.160		0.242		0.205	
Adjusted R^2^ (McFadden)	0.121		0.107		0.190		0.152	

^a^ Respondents who could not be matched to a view (n = 15) are excluded from this analysis.

^b^ Scenario 1: WTT between health benefits in terms of quality of life or life expectancy (yes) or opt out (baseline).

^c^ Scenario 2: WTT between health maximisation or limiting health inequality between patient groups (yes) or opt out (baseline).

^d^ Scenario 3: WTT between children <18 years or elderly >70 years (yes) or opt out (baseline).

^e^ Scenario 4: WTT between patients with a risk of becoming ill due to having an unhealthy lifestyle or due to adversity (yes) or opt out (baseline).

· p-value < 0.10.

* p-value < 0.05.

** p-value < 0.01.

*** p-value < 0.001.

**Table 6:** Impact of background characteristics of traders on preferences for choice of most preferred-option (A or B) in four reimbursement scenarios (logit regression model, weighted data, n presented below table)^a^.

	Scenario 1A^b^		Scenario 2A^c^		Scenario 3A^d^		Scenario 4B^e^	
	B (SE)	OR (95% CI)	B (SE)	OR (95% CI)	B (SE)	OR (95% CI)	B (SE)	OR (95% CI)
Age	0.106 (0.072)	1.111 (0.968,1.287)	-0.088 (0.061)	0.915 (0.811,1.032)	-0.137* (0.066)	0.872 (0.765,0.991)	-0.140* (0.063)	0.870 (0.768,0.982)
Age squared	-0.001 (0.001)	0.999 (0.998,1.0001)	0.001 (0.001)	1.001 (0.999,1.002)	0.001· (0.001)	1.001 (1.000,1.003)	0.001* (0.001)	1.001 (1.000,1.003)
Female	-0.067 (0.313)	0.936 (0.506,1.733)	0.247 (0.296)	1.281 (0.718,2.296)	0.397 (0.304)	1.488 (0.822,2.714)	0.193 (0.310)	1.213 (0.661,2.237)
Education level (low = reference) Middle	1.010* (0.413)	2.745 (1.245,6.351)	0.151 (0.382)	1.163 (0.552,2.486)	0.469 (0.399)	1.599 (0.738,3.553)	0.377 (0.392)	1.458 (0.681,3.192)
High	1.669*** (0.491)	5.309 (2.070,14.322)	0.160 (0.450)	1.174 (0.484,2.853)	1.139* (0.469)	3.122 (1.261,8.000)	0.279 (0.465)	1.322 (0.531,3.312)
Children (no = reference)	0.094 (0.381)	1.098 (0.521,2.331)	-0.230 (0.368)	0.795 (0.382,1.625)	-1.384*** (0.402)	0.251 (0.111,0.539)	-0.224 (0.388)	0.800 (0.370,1.702)
Smoking (not daily = reference)	-0.439 (0.427)	0.644 (0.271,1.464)	-1.343** (0.491)	0.261 (0.090,0.641)	-1.218** (0.467)	0.296 (0.111,0.705)	-1.377** (0.475)	0.252 (0.092,0.607)
View (Equal right to healthcare = reference) Limits to healthcare	1.445*** (0.390)	4.241 (2.012,9.370)	1.236*** (0.365)	3.443 (1.700,7.134)	1.678*** (0.340)	5.354 (2.503,12.088)	1.863*** (0.393)	6.443 (3.050,14.341)
Effective and efficient healthcare	0.514 (0.610)	1.671 (0.494,5.598)	0.971· (0.563)	2.640 (0.879,8.236)	1.049· (0.580)	2.854 (0.921,9.204)	1.399* (0.604)	4.050 (1.283,14.256)
Constant	-4.661* (1.911)	0.009 (0.000,0.357)	1.729 (1.646)	5.633 (0.227,148.336)	3.940* (1.781)	51.411 (1.630,1816,816)	2.401 (1.712)	11.033 (0.394,333.256)
AIC	284.72		318.58		297.68		306.94	
R^2^ (Mc Fadden)	0.229		0.174		0.252		0.205	
Adjusted R^2^ (McFadden)	0.171		0.118		0.198		0.150	

^a^ Respondents who could not be matched to a view (n = 15) are excluded from this analysis.

^b^ Scenario 1A = preference of traders for health benefit in terms of health-related quality of life (n = 105), baseline = respondents who opted out (n = 116).

^c^ Scenario 2A = preference of traders for health maximisation (n = 97), baseline = respondents who opted out (n = 138).

^d^ Scenario 3A = preference of traders for treating children ≤18 years (n = 111), baseline = respondents who opted out (n = 130).

^e^ Scenario 4B = preference of traders for treating persons with a risk of becoming ill due to adversity (n = 102), baseline = respondents who opted out (n = 131).

· p-value < 0.10.

* p-value < 0.05.

** p-value < 0.01.

*** p-value < 0.001.

The results of the LRTs indicated that the overall model could not be significantly improved for any of the scenarios by including additional variables. Hence, logit regression models with the same independent variables are presented for all four scenarios. The VIFs indicated no multicollinearity (VIFs <1.90) for all variables except for age and age squared (VIF 40.78–46.60). The higher VIFs for age and age squared can be explained by the correlation between these two variables. When excluding age or age squared from the regression models, the corresponding VIFs were all <1.62.

In terms of background characteristics, having a higher age (OR 0.845–0.877), having children (OR 0.261), and daily smoking (OR 0.219–0.312) negatively affected, and having a high education level (OR 2.865–3.072) positively affected the WTT of respondents in different reimbursement scenarios. Having a higher age (OR 0.872), having children (OR 0.251), and daily smoking (OR 0.296) were negatively associated, and having a high education level (OR 3.122) was positively associated with a preference for treating children. Having a higher age (OR 0.870) and daily smoking (OR 0.252) were also negatively associated with a preference for treating those with adversity. In addition, daily smoking (OR 0.261) was negatively associated with a preference for health maximisation. Having a middle or high education level (OR 2.745–5.309) was positively associated with a preference for health gains in terms of QOL. Compared to the view “Equal right to healthcare”, the views “Limits to healthcare” (OR 3.306–5.850) and “Effective and efficient healthcare” (OR 2.608–4.375) were positively associated with the WTT of respondents. The view “Limits to healthcare” was also positively associated with a preference for health gains in terms of QOL (OR 4.241), maximising health gains (OR 3.443), treating children (OR 5.354), and those with adversity (OR 6.443). The view “Effective and efficient healthcare” was positively associated with a preference for health maximisation, treating children, and those with adversity (OR 2.640–4.050).

The WTT of respondents with different views on healthcare priority setting differed significantly in each of the scenarios and the majority of non-traders in each of the scenarios consisted of respondents with the view “Equal right to healthcare”. These findings provide evidence in support of hypothesis 1. The logit regression analyses discussed above provide additional evidence in support of hypothesis 1 by indicating that, compared to having the view “Equal right to healthcare”, the views “Limits to healthcare” and “Effective and efficient healthcare” are positively associated with WTT and the most preferred option in all scenarios. The logit regression analyses also provide evidence in support of hypotheses 2 and 3. Having the view “Limits to healthcare” was positively associated with WTT in all scenarios as well as with a preference for health gains in terms of QOL, health maximisation, and treating those with adversity. Having the view “Effective and efficient healthcare” was also positively associated with WTT in all scenarios. In addition, having this view was positively associated with a preference for health maximisation, treating children, and those with adversity. The lower significance levels that accompany these latter associations may be explained by the relatively small number of respondents having the view “Effective and efficient healthcare” (n = 18).

## Discussion

This study was performed against the background of the ongoing debate about societal concerns for an equitable and fair allocation of healthcare resources. The aim of this study was twofold. The first aim was to examine the distribution of three societal views on healthcare priority setting, i.e. “Equal right to healthcare”, “Limits to healthcare”, and “Effective and efficient healthcare” [17], in the general adult population in the Netherlands. The second aim was to examine the relationship between the views and preferences concerning healthcare priority setting, by examining respondents’ WTT between treatments or patient groups in four different reimbursement scenarios as well as by relating respondents’ preferences to background characteristics, including their view.

The results of our study suggest that “Equal right to healthcare” is the most prevalent view on healthcare priority setting in Dutch society. Based on our analyses, we found evidence in support of the hypothesis that respondents with this view had a lower WTT in the different reimbursement scenarios than respondents with the views “Limits to healthcare” and “Effective and efficient healthcare”. In addition, we found evidence in support of hypotheses 2 and 3. The view “Limits to healthcare” is positively associated with WTT and with a preference for health gains in terms of QOL, health maximisation, and treating those with adversity. The view “Effective and efficient healthcare” is positively associated with WTT and with a preference for health maximisation, treating children, and reducing the risk of a life threatening disease for people with adversity. It should be noted, however, that the significance levels of these associations were higher for having the view “Limits to healthcare” than for having the view “Effective and efficient healthcare”. Although, on average, the WTT differed between respondents with different views, our results suggest that the indifference points of those who are willing to trade-off did not differ, hence did not depend on their view. Our results also suggest that those who are willing to trade-off in different reimbursement scenarios generally prefer gains in QOL over LE, maximising health gains over limiting health inequality, treating children over the elderly, and treating those with adversity over those with an unhealthy lifestyle.

The finding that the majority of the general adult population in the Netherlands is willing to trade-off between competing health technologies or patient populations in at least one reimbursement scenario is in line with other empirical studies that suggest that the majority of the public is willing to prioritise in healthcare [4, 23, 24]. Although a large majority was willing to trade-off in at least one scenario, the proportions were close to 50% in each of the scenarios separately. Hence, respondents’ characteristics and their view on healthcare priority setting influenced their WTT and preferences differently in different reimbursement scenarios. As preferences of the public are heterogeneous and may conflict, aligning reimbursement decisions with these preferences is challenging and any allocation decision made by health policy makers may receive opposition from some group in society.

Empirical evidence about the heterogeneity of societal preferences or the relationship between underlying rationales and preferences for priority setting is limited [17, 18, 25–27], and research relating views on healthcare priority setting to such preferences may indeed be considered innovative. Using the same methodology as Wouters et al. [17], Baker et al. [25, 30] identified three views on healthcare priority setting in the UK and examined the distribution of these views in British society. McHugh et al. [26] identified three views on the relative value of end-of-life treatments and, in a more recent study; Mason et al. [27] examined the distribution of these views in British society. Although in both studies, two of the identified views share similarities with the views “Equal right to healthcare” and “Effective and efficient healthcare” of Wouters et al. [17], none of the views appeared to be as dominant in the UK [25, 27] as the view “Equal right to healthcare” in the Netherlands. Van Exel et al. [18] identified five views on healthcare priority setting in ten European countries, among which the Netherlands and the UK. Mason et al. [31] examined the distribution of these views in a subset of nine countries. The results of this study support our finding that an egalitarian view on healthcare priority setting is the most prevalent view in the Netherlands. In addition, the results of this study suggest that an egalitarian view on healthcare priority setting in the most prevalent view in the UK as well as in the other European countries. Further comparative research will be necessary to investigate the difference in views and their distribution between countries, and the relationship between the views and societal preferences for priority setting in these countries. The results of our study generally align with the results of other studies indicating that *the* social value of the QALY does not exist [14] as societal concerns for an efficient and equitable allocation of health and healthcare are heterogeneous. Our findings support those of other studies indicating, for example, that priority should be given to younger over older people (e.g. [32–36]) and to those with adversity over those with an unhealthy lifestyle [37–39].

Some limitations of this study need to be mentioned. A first limitation concerns the four relatively simple WTT exercises. Because our primary aim was to explore the relationship between the three societal views and preferences in a number of reimbursement scenarios based on distinguishing characteristics of those views, we chose for fairly straightforward WTT exercises. In addition, we expected that respondents might find the WTT exercises rather difficult and, therefore, kept the WTT exercises clear and concise. A second limitation concerns the initial lack of representativeness of our sample, resulting from suboptimal recruitment of respondents. To improve our sample’s representativeness, we weighted the data by applying a combined weighting factor for age, gender, and education level. Although this method is often associated with an increased level of uncertainty concerning the results, a comparison of the results pre and post weighting indicated no major changes in the size or direction of estimates. A third limitation concerns the non-randomised order in which the reimbursement scenarios were presented to respondents. However, as respondents were presented with only four scenarios that differed in terms of treatment and patient characteristics, we expect the possible risk of order bias to be limited. A fourth limitation is concerned with the possibility for respondents to avoid prioritisation and opt out in each of the four scenarios. Although we provided an opt-out to examine respondents’ WTT and to explore non-traders’ reasons for opting out in each of the scenarios, in decision-making practice opting out is not possible for health policy makers. It is unclear, how not providing an opt-out would have influenced the results of our study. A fifth limitation concerns the exclusion of respondents that could not be matched from the logit regression analyses. These respondents significantly differed in age and nationality from respondents who could be matched and, excluding these respondents resulted in a loss of information concerning our sample’s preferences in the different reimbursement scenarios. However, excluding these respondents did not affect our primary aim of conducting the regression analyses, i.e. to explore the relationship between the three views and respondents’ preferences in different reimbursement scenarios. In addition, as the excluded group of respondents was relatively small (n = 15) and could not be matched to one of the views, we considered the loss of information to be limited. A final limitation concerns the lack of a normative discussion of the views and preferences concerning healthcare priority setting. Our aim was to examine the distribution of the three views and the relationship between the views and preferences and, therefore, a normative discussion was outside the scope of this paper. We refer the interested reader to e.g. Schwappach [4], Olsen et al. [15], Ottersen [40], and Bognar [41, 42] for normative discussions about societal preferences concerning healthcare priority setting. In addition to these limitations, we would like to address that we consider it a strength of our study that we combined different methods to examine the relationship between societal views on healthcare priority setting and preferences in different healthcare reimbursement scenarios. This type of study is regarded as methodologically challenging [30] and is infrequently conducted.

Our results indicate that societal preferences concerning healthcare priority setting are heterogeneous and complex as people’s view on healthcare priority setting and background characteristics influence their preferences differently in different reimbursement scenarios. Hence, when aiming to align allocation decisions with societal preferences for equity and efficiency, the use of a mix of equity concerns in decision-making practice is recommended. As we examined the relationship between societal views and preferences in the context of only four reimbursement scenarios, we recommend extending our research to scenarios that include other potential sources that contribute to the social value of the QALY [4, 14]. For example, patients’ prior healthcare consumption, the duration of health benefits, and the burden of illness that is associated with a disease. As heterogeneous preferences may sometimes be conflicting, aligning allocation decisions with societal preferences is challenging and decisions will almost inevitably receive opposition from some group or another in society. Given the available evidence, this is unlikely to be a strictly Dutch phenomenon. Hence, knowledge about the (distribution of the) societal views and related preferences concerning healthcare priority setting may help health policy makers to be considerate of these views and preferences when allocating resources in healthcare. This knowledge, for example about the high prevalence of the egalitarian view on healthcare priority setting, may also help health policy makers in communicating and explaining (inevitable) allocation decisions to the public.

## Conclusions

The results of this study suggest that “Equal right to healthcare” is the most prevalent view on healthcare priority setting in the Netherlands. Although we expected this egalitarian view to be negatively associated with WTT, our results indicate that the majority of people is still willing to prioritise between competing health technologies or patient groups regardless of their view on healthcare priority setting. People’s characteristics and views on healthcare priority setting influence preferences differently in different reimbursement scenarios. As societal views and preferences are heterogeneous and may conflict, aligning allocation decisions with societal preferences remains challenging and any decision may be expected to receive opposition from some group in society. When aiming to align allocation decisions with societal preferences concerning healthcare priority setting, accounting for the variety in societal views and preferences is recommended.

## Supporting information

S1 File Example of reimbursement scenario. (DOCX)

S2 File Dataset of the study variables. (CSV)
