# Supplementary material for: Who should receive treatment? An empirical enquiry into the relationship between societal views and preferences concerning healthcare priority setting
Source: PLoS One. 2018 Jun 27;13(6):e0198761. doi: 10.1371/journal.pone.0198761 (PMC6021057; doi:10.1371/journal.pone.0198761)
Supplement: S1 File — (DOCX) [file pone.0198761.s001.docx]

**S1 File. Example of reimbursement scenario**

General introduction:

Healthcare resources are scarce. Therefore, health policy makers have to decide which treatments can be reimbursement from the public health insurance package, and which cannot. These decisions are often difficult, as reimbursing treatment for one patient group implies not being able to reimburse (the best possible) treatment for another.

Presented below are some of these difficult decisions. Which decision would you advise health policy makers to make in these scenarios? Which choice do you consider best in order to allocate healthcare resources as optimal as possible?

**Scenario 1**

Introduction:

There are two possible treatments for patients with a specific type of cancer. Only one of these treatments can be reimbursed. Without treatment, the patients have a life expectancy of 3 months and their quality of life is 3 on a scale from 0 to 10. The ‘0’ represents the worst health possible and ‘10’ represents the best health possible.

**Question 1:**

Which treatment would you advise health policy makers to choose?

- Treatment A improves patients’ quality of life with 3 points (from 3 to 6), but does not influence their life expectancy.
- Treatment B improves patients’ life expectancy with 3 months (from 3 to 6), but does not influence their quality of life.
- I do not have a preference for treatment A or treatment B.

**Question 2:**

When choosing treatment A:

Suppose that the improvement in quality of life by treatment A is unsatisfactory and less than 3 points. According to you, at which point will treatment A (improvement in quality of life) be equally good as treatment B (improvement in life expectancy of 3 months)?

- An improvement in quality of life of 2.5 points is equally good as an improvement in life expectancy of 3 months.
- An improvement in quality of life of 2.0 points is equally good as an improvement in life expectancy of 3 months.
- An improvement in quality of life of 1.5 point is equally good as an improvement in life expectancy of 3 months.
- An improvement in quality of life of 1.0 point is equally good as an improvement in life expectancy of 3 months.
- An improvement in quality of life of 0.5 point is equally good as an improvement in life expectancy of 3 months.
- An improvement in quality of life of 0 points is equally good as an improvement in life expectancy of 3 months.

When choosing treatment B:

Suppose that the improvement in life expectancy by treatment B is unsatisfactory and less than 3 months. According to you, at which point will treatment B (improvement in life expectancy) be equally good as treatment A ((improvement in quality of life of 3 points)?

- An improvement in life expectancy of 2.5 months is equally good as an improvement in quality of life of 3 points.
- An improvement in life expectancy of 2.0 months is equally good as an improvement in quality of life of 3 points.
- An improvement in life expectancy of 1.5 month is equally good as an improvement in quality of life of 3 points.
- An improvement in life expectancy of 1.0 month is equally good as an improvement in quality of life of 3 points.
- An improvement in life expectancy of 0.5 month is equally good as an improvement in quality of life of 3 points.
- An improvement in life expectancy of 0 months is equally good as an improvement in quality of life of 3 points.

When choosing the opt-out:

Why do you not have a preference for treatment A or treatment B?

- Both treatments are equally effective for these patient groups.
- Both treatments are equally ineffective for these patients groups.

Other: …
